# A peptide derived from interleukin-10 exhibits potential anticancer activity and can facilitate cell targeting of gold nanoparticles loaded with anticancer therapeutics

**DOI:** 10.1038/s42004-023-01079-x

**Published:** 2023-12-15

**Authors:** Chun-Chun Chang, Chin-Hao Yang, Chin-Hsien Chuang, Shinn-Jong Jiang, Yin-Min Hwang, Je-Wen Liou, Hao-Jen Hsu

**Affiliations:** 1 Department of Laboratory Medicine, Hualien Tzu Chi Hospital, Buddhist Tzu Chi Medical Foundation, Hualien, 97004 Taiwan, ROC; 2 Department of Laboratory Medicine and Biotechnology, College of Medicine, Tzu Chi University, Hualien, 97004 Taiwan, ROC; 3 Department of Biochemistry, School of Medicine, Tzu Chi University, Hualien, 97004 Taiwan, ROC; 4 Department of Biomedical Sciences and Engineering, College of Medicine, Tzu Chi University, Hualien, 97004 Taiwan, ROC

**Keywords:** Peptides, Biophysical chemistry, Drug delivery, Lead optimization, Cheminformatics

## Abstract

Human interleukin-10 (IL-10) is an immunosuppressive and anti-inflammatory cytokine, and its expression is upregulated in tumor tissues and serum samples of patients with various cancers. Because of its immunosuppressive nature, IL-10 has also been suggested to be a factor leading to tumor cells’ evasion of immune surveillance and clearance by the host immune system. In this study, we refined a peptide with 20 amino acids, named NK20a, derived from the binding region of IL-10 on the basis of in silico analysis of the complex structure of IL-10 with IL-10Ra, the ligand binding subunit of the IL-10 receptor. The binding ability of the peptide was confirmed through in vitro biophysical biolayer interferometry and cellular experiments. The IL-10 inhibitory peptide exerted anticancer effects on lymphoma B cells and could abolish the suppression effect of IL-10 on macrophages. NK20a was also conjugated with gold nanoparticles to target the chemotherapeutic 5-fluorouracil (5-FU)-loaded nanoparticles to enhance the anticancer efficacy of 5-FU against the breast cancer cell line BT-474. Our study demonstrated that NK20a designed in silico with improved binding affinity to the IL-10 receptor can be used as a tool in developing anticancer strategies.

## Abstract (web summary)

Human interleukin-10 (IL-10) is known to be an immunosuppressive cytokine for anti-inflammation, however, IL-10 can enable the evasion of tumor cells through immunosuppression of the host immune system. Here, the authors develop an IL-10 inhibitory peptide, NK20a, that displays anticancer effects, and show that its conjugation with chemotherapeutic gold nanoparticles enhances their anticancer efficacy.

## Introduction

Interleukin-10 (IL-10) is an immune regulatory cytokine with strong anti-inflammatory activity^1^. It is produced by various cells, including T cells, B cells, keratinocytes, mast cells, eosinophils, dendritic cells, monocytes, and macrophages^1,2^. However, IL-10 has also been associated with carcinogenesis. Both pro- and antitumor effects of IL-10 have been proposed^3–5^. As an antitumor factor, IL-10 has been suggested to directly induce the expansion of tumor-specific CD8^+^ T cells in the tumor and cytotoxic activity of these cells^4^. IL-10 was also found to promote T-cell memory and upregulation of INF-γ expression^4,6,7^. In the antitumor aspect, PEGylated IL-10 was tested for anticancer applications of IL-10, and some degree of favorable outcomes was observed^8^. By contrast, the positive correlation between cancer and IL-10 has attracted considerable interest, and IL-10 is considered a cancer biomarker and target for the development of cancer therapies. It has been suggested that IL-10 suppresses macrophage and proinflammatory Th17 T-cell responses to dampen the immune response to cancer^4^. IL-10 also promotes tumor cell proliferation and metastasis through immunosuppression^9,10^, and IL-10-mediated immunosuppression is linked to the synthesis of tumor necrosis factor, IL-1, IL-12, and chemokines and the downregulation of the surface costimulatory molecule CD80/86 on tumors^10^. Notably, IL-10 expression levels were significantly higher in the tumor tissues and serum samples of patients with cancer^1,11–14^. In patients with breast cancer, elevated levels of IL-10 were observed in serum, which is closely related to tumor progression^15^. Moreover, the expression of IL-10 and its receptor is regulated by autocrine IL‑10 signaling pathways^16–18^. Therefore, IL-10 antagonists may be a critical factor that influences the success of cancer treatments.

The IL-10 receptor is a tetrameric type II cytokine receptor composed of two ligand-binding subunits (IL-10Ra) and two signaling subunits (IL-10Rb). The structure of IL-10 forming a complex with IL-10Ra has been studied experimentally through X-ray crystallography and electron microscopy^19,20^, allowing us to analyze the interactions between IL-10 and its receptor. We previously analyzed the complex structure of IL-10 and designed a peptide with 25 amino acids from the binding region of IL-10^21^. This peptide could specifically bind to IL‑10Ra on the cell surface of two B‑lineage cell lines, the lymphoma B cell line (BJAB) and lymphoblastoid cell line.

Conventional strategies for the therapeutic administration of anticancer drugs usually have adverse side effects induced by the ubiquitous distribution of the chemotherapeutics throughout the body, with an insufficient amount of the drug reaching the specific site of treatment. To overcome this limitation, the use of drug delivery carriers has been proposed^22^. Gold nanoparticles present numerous advantages for this purpose, such as easy surface modification, unique optical properties, and good biocompatibility, and therefore have been used to deliver chemotherapeutic drugs to cancer cells and for diagnostic purposes^23–25^.

In this study, using in silico approaches, we designed a peptide with superior binding affinity to IL-10Ra that can be used to inhibit the effects of IL-10. The functions of the peptide were examined using cellular experiments. We also conjugated the peptide with gold nanoparticles to explore the possibility of using the peptide to navigate the anticancer drug-loaded nanoparticles to cancer cells and enhance the anticancer therapeutic efficacy of the drug.

## Materials and methods

### Molecular docking of designed peptides to IL-10Ra

In our previous study^21^, the surface charge distribution of the binding interface between IL‑10 and IL‑10Ra showed that electrostatic interactions might dominate the IL‑10 binding to the extracellular domain of IL‑10Ra. On the basis of the complex structure of IL-10 and IL-10Ra, a peptide NM25, derived from the binding sequence of IL-10 to IL-10Ra, was designed and synthesized to inhibit the binding of IL-10 to its receptor IL-10Ra. In this study, we modified the sequence with fewer residues and higher binding ability to IL-10Ra. Two peptides, namely NK20 and NK20a, were designed in this study. Table 1 presents the sequences of the original NM25 peptide, the two newly designed peptides NK20 and NK20a, and the negative control peptide for the experiments. NK20 is a truncation of NM25, and NK20a is a modified form of NK20 with two amino acid residues altered. Due to that the electrostatic interactions are crucial in the binding of IL-10 to IL-10Ra, we tried to preserve the positively charged residues and reduce the negatively charged residues by replacing Asp (D) with Asn (N). To predict the preferable binding sites of the designed peptides to the extracellular domain of IL-10Ra, molecular docking and structural analysis were performed using the docking module of the Molecular Operating Environment software package (MOE2020.09; http://www.chemcomp.com). The molecular docking was performed by using the “*Protein-Protein Dock*” module in the MOE software package. “*Triangle Matcher*” was used for the placement method, and the “*Affinity ΔG scoring function*” was used to score the top 10,000 poses obtained from placement. From these poses, the top 100 poses were then refined via the explicit molecular mechanics forcefield method, where energy minimization of the system was performed, and charges of all atoms were reassigned using the AMBER10: EHT forcefield. From the remaining 100 poses, 30 were chosen and rescored with the “*GBVI/WSA ΔG scoring function*” to estimate the binding free energy of each pose.

**Table 1:** Sequences of designed peptides.

No	Type	Name	Peptide sequence
1	original	NM25	NMLRDLRDAFSRVKTFFQMKDQLDN
2	refined	NK20	NMLRDLRDAFSRVKTFFQMK
3	refined	NK20a	NMLRNLRNAFSRVKTFFQMK
4	random	CG20	CPLNGSTVYGHLRHCLSCSG

### Molecular dynamics simulations and MM/PBSA binding free energy calculations

All molecular dynamics (MD) simulation protocols used in this study were performed using the GROMACS-2018 software package with a GROMOS54A7 force field and an integration step size of 2 fs. A 50-ns MD simulation was conducted with no constraint on the complex structure after energy minimization and equilibration. The simulations were conducted in the NPT ensemble by employing the velocity-rescaling thermostat at a constant temperature of 310 K and pressure of 1 bar. The complex protein and solvent were separately coupled with a temperature coupling time of 0.1 ps. The systems were neutralized with sodium and chloride ions to simulate the condition of 0.15 M NaCl solution. Isotropic pressure coupling was applied with a coupling time of 0.1 ps and a compressibility of 4.5 × 10^−5^ bar^−1^ for the x, y, and z directions. Long-range electrostatic force was calculated using the particle-mesh Ewald summation algorithm with grid dimensions of 0.12 nm and an interpolation order of 4. The cutoffs for the Lennard–Jones and short-range Coulomb interactions were set as 1.4 and 1.0 nm, respectively. The simulation protocols have been detailed in our previous studies^21,26–29^.

To determine the most stable binding poses of the designed peptides to receptor IL-10Ra predicted by molecular docking, the binding free energy (Δ*G*_*bind*_) was estimated for each complex by using the MM/PBSA approach on the basis of the snapshots extracted from the single trajectory of the complex (single trajectory method), which was implemented in GROMACS, a GMXAPBS tool developed by the Musco group^30^.

### Peptide synthesis

All the designed peptides in this study were chemically synthesized using GeneMark (GMbiolab Co., Ltd., Taiwan) and a solid-phase methodology. The molecular weights and purities of the synthetic peptides were confirmed using high-performance liquid chromatography and electrospray ionization mass spectrometry. The purity of peptides was over 95% (Fig. S1).

### Cell culture

BJAB B-lymphoma cell lines were cultured in RPMI 1640 medium supplemented with 10% fetal bovine serum. RAW 264.7 macrophage and BT-474 human breast tumor cell lines were cultured in Dulbecco’s Modified Eagle Medium (DMEM) supplemented with 10% fetal bovine serum, 1% L-glutamine, 100 U/ml penicillin, 100 U/ml streptomycin, and sodium pyruvate. The cells were incubated at 37 °C in a 5% CO_2_ atmosphere.

### Biolayer interferometry kinetic assay

Biolayer interferometry (BLI) was applied to analyze peptide–receptor protein interactions. BLI was performed with a ForteBio Octet RED96e System (Sartorius AG, Göttingen, Germany) by using high-precision streptavidin and biosensors. The biosensors were hydrated in a plate for 15 min and then loaded with 10 μg/mL recombinant receptor human IL-10Ra protein and incubated for 150 s. The associations with the designed peptides at concentrations of 3.12, 6.25, 12.5, 25.0, 50.0, and 100.0 μM were observed for 120 s. After the association step, the biosensor was shifted to the dissociation buffer, phosphate-buffered saline (PBS) with 0.05% Tween20, for dissociation. Dissociation was recorded until equilibrium was reached (120 s).

### Peptide binding assay

To assess the binding ability of the designed peptide to the IL-10 receptor on the cell surface, 50 μg/mL NK20a-FITC peptide was treated with 1 × 10^5^ of lymphoma B cells at room temperature for 1 h. Following two washes with PBS, the cells were resuspended in 500 μL of PBS, and peptide-cell binding was analyzed through fluorescent flow cytometry (CytoFLEX, Beckman Coulter, CA, USA). A total of 1 × 10^4^ cells were acquired to experimentally measure cell fluorescence. Viable cells were gated based on cell size and granularity. CytEXpert software (Beckman Coulter, CA, USA) was used for data collection and analysis.

### Cell viability assay

For the cell viability assay, B-lymphoma cells were cultured in 96-well plates at a density of 3 × 10^3^ cells/well for 24 h. The cells were then treated with 10 ng/mL IL-10 and 50 μg/mL NK20a, and further incubated for 48 h. After incubation, 10 μL of Cell Counting Kit-8 (CCK-8; Enzo Life Sciences, NY, USA) reagent was added, and the cells were incubated at 37 °C for 1 h. Cell viability was quantified by measuring the absorbance at 450 nm by using an ELISA plate reader (CLARIOstar Plus, BMG Labtech, Ortenberg, Germany).

### Atomic force microscopy

Cell morphology was evaluated through atomic force microscopy (AFM) to observe the effects of the peptide on the cells. For AFM experiments, RAW 264.7 macrophages (5 × 10^4^ cells/mL) were seeded on coverslips that had been treated with HCl to facilitate cell attachment. The cells were treated with lipopolysaccharides (LPS), NK20a, or IL-10 and incubated at 37 °C. After 24 h of incubation, the medium was removed, and the cells were washed twice with PBS. The cells were then fixed with 4% glutaraldehyde for 40 min. Finally, the fixation solution was removed, the samples were gently washed three times with Milli Q water, and then the samples were dried in a desiccator for 3 h. Cell morphology was imaged using an AFM instrument (Nanowizard, JPK BioAFM, Bruker Nano GmbH, Berlin, Germany). Contact-mode scanning was used for imaging at scanning rates of 0.5–1.5 Hz and a scanning resolution of 512 × 512 pixels. The AFM probes used were oxide-sharpened silicon nitride (Si_3_N_4_) probes (OMCL-TR400PB-1, Olympus, Tokyo, Japan) with a spring constant of 0.02 N/m. The SPM image processing software package version 3.1.6 (JPK BioAFM, Bruker Nano GmbH, Berlin, Germany) was used for processing and analysis of AFM image data.

### Fabrication of NK20a@Au and NK20a/Poly A15@Au

We next conjugated the peptides to gold nanoparticles. For the preparations, streptavidin-conjugated gold nanoparticles were purchased from Abcam Co. (ab186864, Cambridge, UK). NK20a-conjugated gold nanoparticles (NK20a@Au) and NK20a/poly 15 adenine co-conjugated gold nanoparticles (NK20a/Poly A15@Au), were fabricated through streptavidin–biotin interactions. Poly A15 was used in this study to load 5-FU onto the nanoparticles. The 15-adenine polynucleotides are ideal for base pairing the anticancer drug 5-FU with hydrogen bonds, and the drug can be gradually released in physiological environments^31^. Streptavidin-conjugated gold nanoparticles were centrifuged at 9000 × *g* for 10 min and resuspended in Milli Q water to a suspension optical density of 2.5 at 530 nm. Biotinylated-NK20a (100 μM) and Biotinylated-Poly A15 (100 μM) were dissolved in 100 μL of Milli Q water and were added to the streptavidin-conjugated gold nanoparticle solution accordingly. The mixtures were incubated for 2 h at 4 °C. After incubation, the nanoparticles were centrifuged, and the pellets were washed three times with Milli Q water. We also loaded the anticancer drug 5-fluorouracil (5-FU) onto the gold nanoparticles. The 5-FU loading capacity of the nanoparticles was determined as follows: 5-FU solution (500 µM) was added to the NK20a/Poly A15@Au nanoparticle suspension. After 24 h of incubation at 4 °C, the suspension was centrifuged at 9000 × *g* for 10 min to pellet down the nanoparticles. The absorbance of the supernatant was measured at 266 nm to determine the amount of nonconjugated 5-FU; the amount of 5-FU was determined by deducting the amount of nonconjugated 5-FU from the total 5-FU added. The 5-FU-loaded NK20a/Poly A15@Au nanoparticles were centrifuged at 9000 × *g* for 10 min, and the pellets were washed thrice with Milli Q water. Finally, the nanoparticles were subjected to Fourier-transform infrared (FTIR) spectroscopy. The prepared peptide/5-FU loaded nanoparticles were ready to be used in subsequent investigations.

### Characterization of peptide-conjugated gold nanoparticles

The functionalized nanoparticles of NK20a@Au were characterized using attenuated total reflection FTIR spectroscopy (ATR-FTIR). For the FTIR analysis, the samples were placed on top of a crystal sample holder and dried in a desiccator. An average of 48 scans were conducted for each spectrum with a nominal resolution of 1 cm^−1^ and a wavelength range of 4000–600 cm^−1^. The hydrodynamic diameter and polydispersity index of the gold nanoparticles were determined through dynamic light scattering (DLS), and the zeta potential of the particles was measured using laser Doppler velocimetry with an ELSZ-2000ZS instrument (Otsuka Tech Electronics, Osaka, Japan).

### Evaluation of cellular uptake of gold nanoparticles under a confocal microscope

B-lymphoma cells (5 × 10^5^ cells/mL) were seeded in Shi-fix coverslips (Shikhar Biotech) and incubated in RPMI 1640 medium supplied with 10% FBS under a 5% CO_2_ atmosphere. After 24 h of incubation, the cells were washed twice with PBS and treated with FITC-labeled NK20a peptide (NK20a-FITC) or NK20a-FITC@Au nanoparticles for 1 h at 37 °C. For visualization, the cell membranes were stained with Cytopainter (ab219942, Abcam, Cambridge, UK), and the nuclei were stained with 4’,6-diamidino-2-phenylindole (DAPI). The samples were each treated with 100 µL of Cytopainter for 20 min, then washed with fresh PBS. Subsequently, the cells were fixed with 4% glutaraldehyde for another 20 min. After the fixation, 3–4 drops of mounting medium with 0.0002% DAPI (ab104139, Abcam, Cambridge, UK) were applied directly to the samples to stain the nuclei of cells. The prepared samples were imaged with a confocal fluorescence microscope (C2si + , Nikon, Tokyo, Japan).

### Evaluation of therapeutic efficacy of loaded gold nanoparticles

For the evaluation of therapeutic efficacy of the loaded gold nanoparticles, BT-474 breast cancer cells were plated onto 96-well plates at a density of 5 × 10^3^ cells/well in DMEM supplemented with 10% FBS and incubated for 24 h at 37 °C. After 24 h, the cells were treated with 5-FU, NK20a/Poly A15@Au, Poly A15 + 5-FU@Au or NK20a/Poly A15 + 5-FU@Au for another 48 h. After treatment, 10 μL of the CCK-8 reagent (Dojindo, Japan) was added, and the cells were incubated for 1 h at 37 °C. Cell viability was determined by measuring the absorbance at 450 nm by using an ELISA plate reader (CLARIOstar Plus, BMG Labtech, Ortenberg, Germany).

### Reporting summary

Further information on research design is available in the Nature Portfolio Reporting Summary linked to this article.

## Results

### Binding free energy calculations for the designed peptides docked to receptor IL-10Ra

**Fig. 1:**
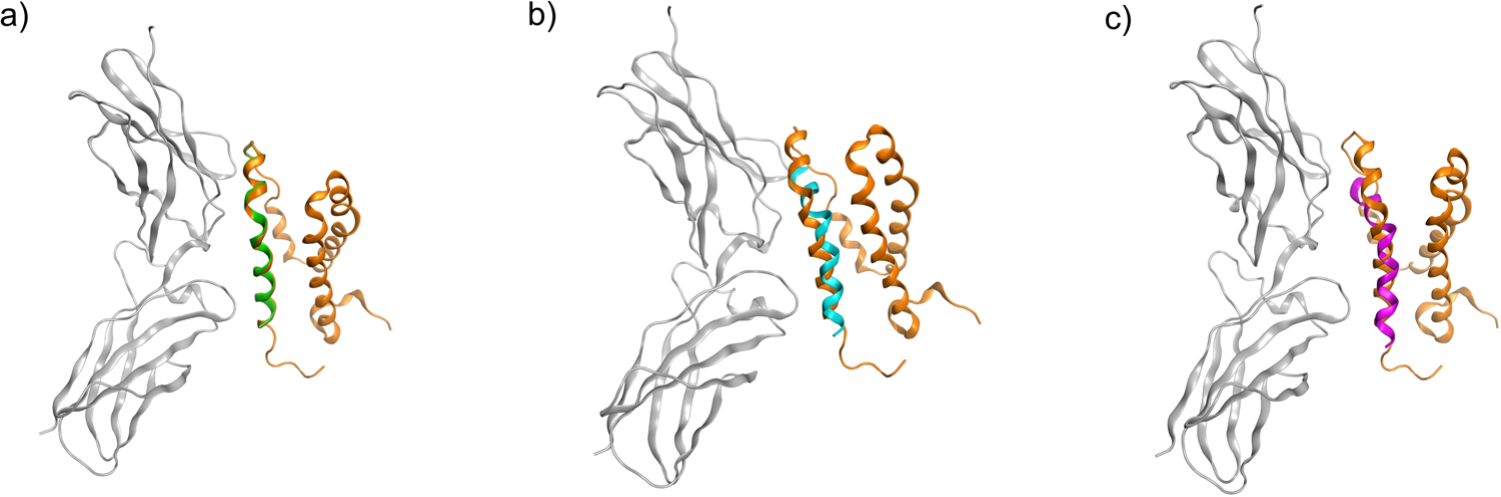
Superposition of designed peptides with the complex structure of IL-10 bound to IL-10Ra. The subunit receptor IL-10Ra is shown in gray color; IL-10 is shown in orange color. **a** Originally designed peptide NM25 (green color). **b** Refined peptide NK20 (cyan color). **c** Refined peptide NK20a (purple color).

**Fig. 2:**
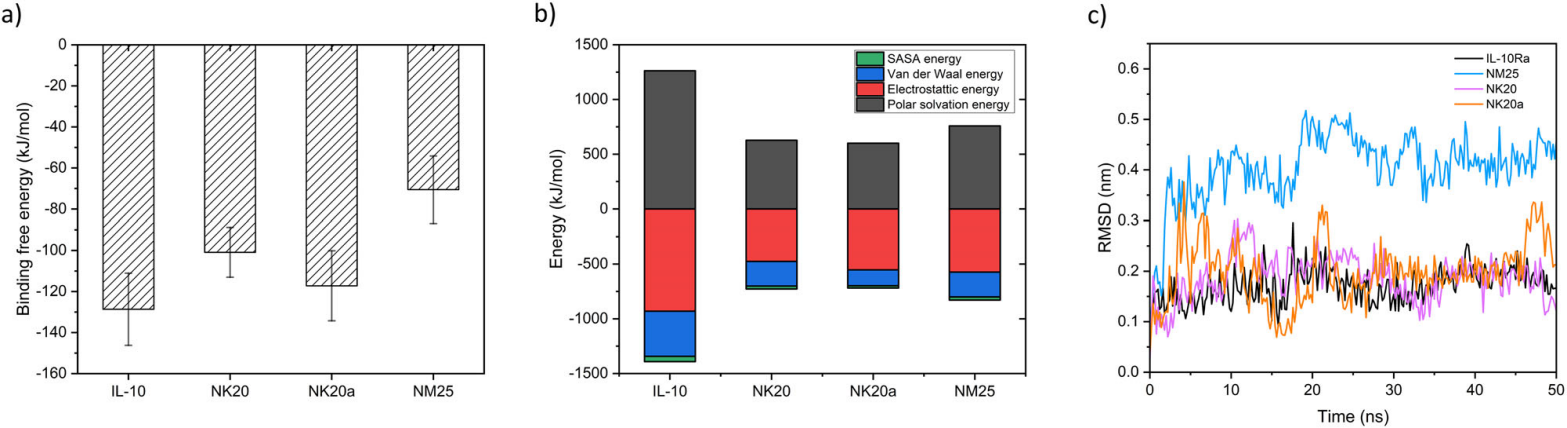
MM/PBSA binding free energy calculations for designed peptides docked to receptor IL-10Ra. **a** IL-10 and three designed peptides bound to receptor IL-10Ra. The binding energy of NK20a is lower than that of the original peptide NM25. A total of 200 snapshots extracted from the last 20 ns stable MD trajectory per system were used to perform MM/PBSA. Error bars represent the standard deviation of calculations. **b** Detailed analysis of the components of binding free energies shows that electrostatic interactions dominate the binding (red color), followed by the solvation free energies (black color) and van der Waals (VDW) interactions (blue color). **c** RMSD profiles with simulation time for the designed peptides and receptor IL-10Ra.

We previously designed a 25-amino-acid peptide (NM25) based on the molecular docking of IL-10 to its receptor IL-10Ra. This peptide could specifically bind to receptor IL‑10Ra on the cell surface of BJAB^21^. In the present study, we refined this peptide to achieve stronger binding to the receptor IL-10Ra. Two additional peptides (NK20 and NK20a) were designed in silico based on the NM25 sequence. These three peptides (NM25, NK20, and NK20a) were docked to IL-10Ra (Fig. 1) through the molecular docking approach, and the MM/PBSA binding free energies of the preferable binding poses of the three peptides to IL-10Ra were calculated for comparison (Fig. 2a, b). The results revealed that the binding of NK20a to IL-10Ra entailed the lowest free energy (−118 kJ/mol) among the three peptides. Root mean square deviation (RMSD) profiles and simulation times revealed that the refined peptides NK20 and NK20a had lower RMSD values than the originally designed peptide NM25, indicating the interactions between the refined peptides and the receptor IL-10Ra are more stable than the original one (Fig. 2c). The snapshots of NM25 bound to receptor IL10Ra at different simulation times also showed that NM25 has larger structural changes after 5 ns simulation time while NK20a maintained the helical structure during the whole simulation time (Fig. S2). An analysis of the MM/PBSA binding free energies for these three peptides also revealed that electrostatic interactions dominated the binding of NK20a to IL-10Ra.

### Confirmation of binding of designed peptides to IL-10Ra

**Fig. 3:**
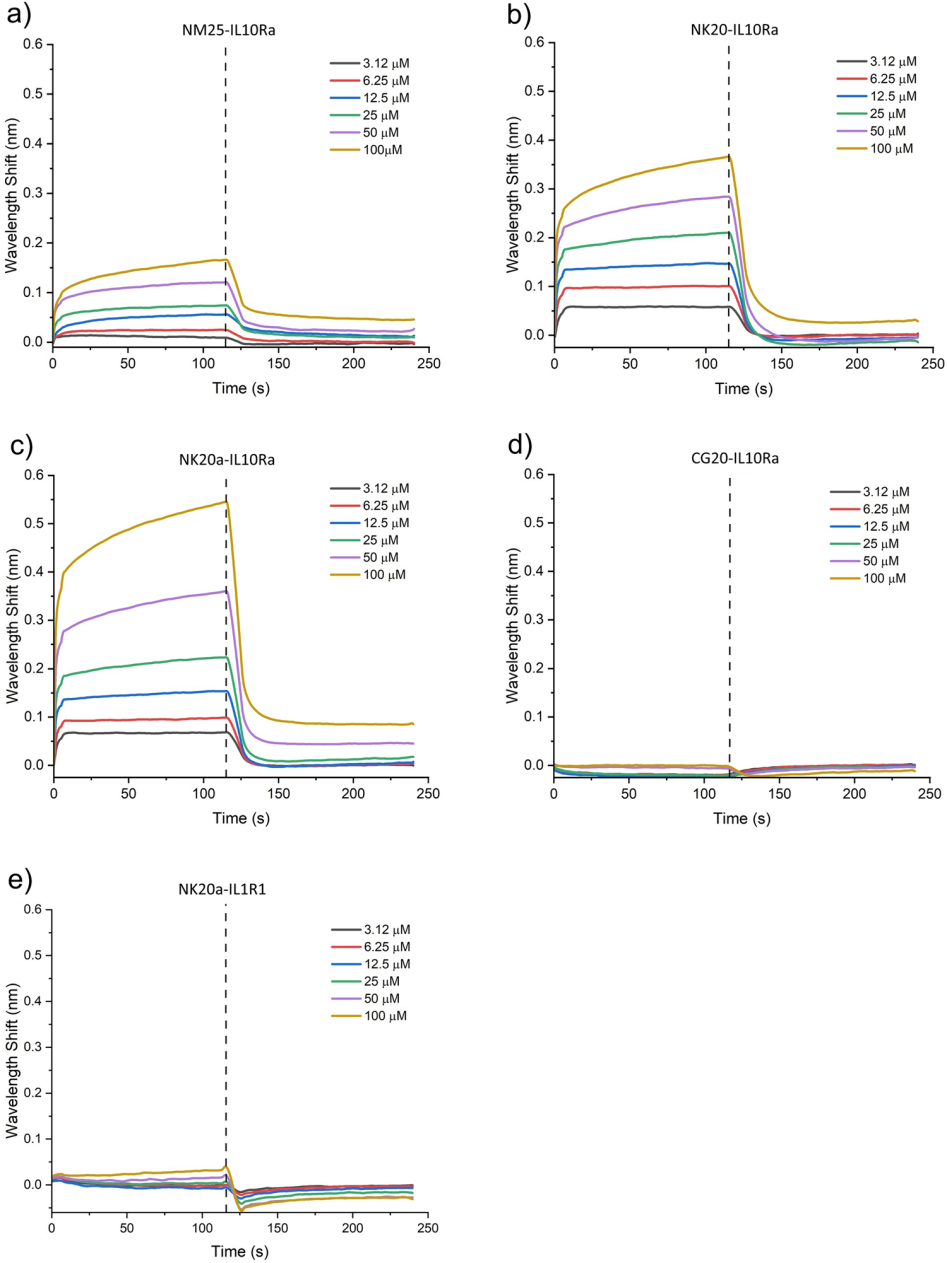
BLI kinetic assays of the designed peptides binding to the receptor IL-10Ra. Wavelength shift curves show the association and dissociation of the designed peptides (**a**) NM25, (**b**) NK20, (**c**) NK20a, (**d**) CG20 binding to the IL-10 receptor, and (**e**) NK20a binding to a control receptor IL-1R1 at various concentrations. Dissociation was recorded at 120 s after the binding started (black dotted line).

To measure the binding ability of the designed and negative control peptides (NM25, NK20, NK20a, and control scramble peptide CG20) to the receptor IL-10Ra in vitro, BLI experiments were performed. The results of BLI experiments are presented in Table 2, Fig. 3 and S3. According to the results, both the refined peptides NK20 and NK20a exhibited higher binding affinity to IL-10Ra than did the original peptide NM25. When binding with IL-10Ra, the association constant (k_a_) of NK20 and NK20a was 1.02 × 10^4^ (1/Ms) and 8.14 × 10^3^ (1/Ms), respectively. The k_a_ values of the refined peptides were approximately 12-fold higher than that of NM25. Further, NK20a had a very low dissociation constant (k_d_) when binding to IL-10Ra (0.09088 (1/s)), indicating a stronger binding of NK20a to the receptor as compared to that of NK20 (0.2185 (1/s)) (Table 2). In order to examine the specific binding of NK20a to IL-10Ra, we applied a negative control peptide to interact with IL-10Ra and found that the scramble peptide CG20 was not able to bind to IL-10Ra in the BLI experiments (Fig. 3d). We also measured the binding of NK20a to receptor IL-1R1, which is suggested not to be a receptor for IL-10, and the results are shown in Fig. 3e. As can be seen in Fig. 3e, the binding of NK20a to IL-1R1 was poor. The equilibrium dissociation constant (K_D_) of NK20a interactions with IL-1R1was approximately 2000 times greater than that with IL-10Ra, and the calculated dissociation constant of NK20a interactions with IL-1R1 was to be 5000 times greater than that with IL-10Ra interactions (Table 2). These results indicated that the binding between NK20a and IL-10Ra is specific. Of the designed peptides, NK20a exhibited the strongest binding affinity to IL-10Ra, with the lowest K_D_ of approximately 1.12 × 10^−5^. As a result, NK20a was used for further investigations in this study.

**Table 2:** Association and dissociation constants of designed peptides binding to the receptor IL-10Ra.

Ligand	Receptor	KD (M)	ka (1/Ms)	kd (1/s)
IL-10	IL-10Ra	<1.0 × 10^−12^	2.24 × 10^5^	<1.0 × 10^−7^
NM25	IL-10Ra	4.1 × 10^−5^	8.32 × 10^2^	0.0341
NK20	IL-10Ra	2.14 × 10^−5^	1.02 × 10^4^	0.2185
NK20a	IL-10Ra	1.12 × 10^−5^	8.14 × 10^3^	0.09088
CG20	IL-10Ra	1.4 × 10^16^	1.49 × 10^−6^	2.1 × 10^10^
NK20a	IL-1R1	2.8 × 10^−2^	1.83 × 10^4^	512

### Binding of the refined peptide NK20a to the cell surface and its inhibition of cell proliferation

**Fig. 4:**
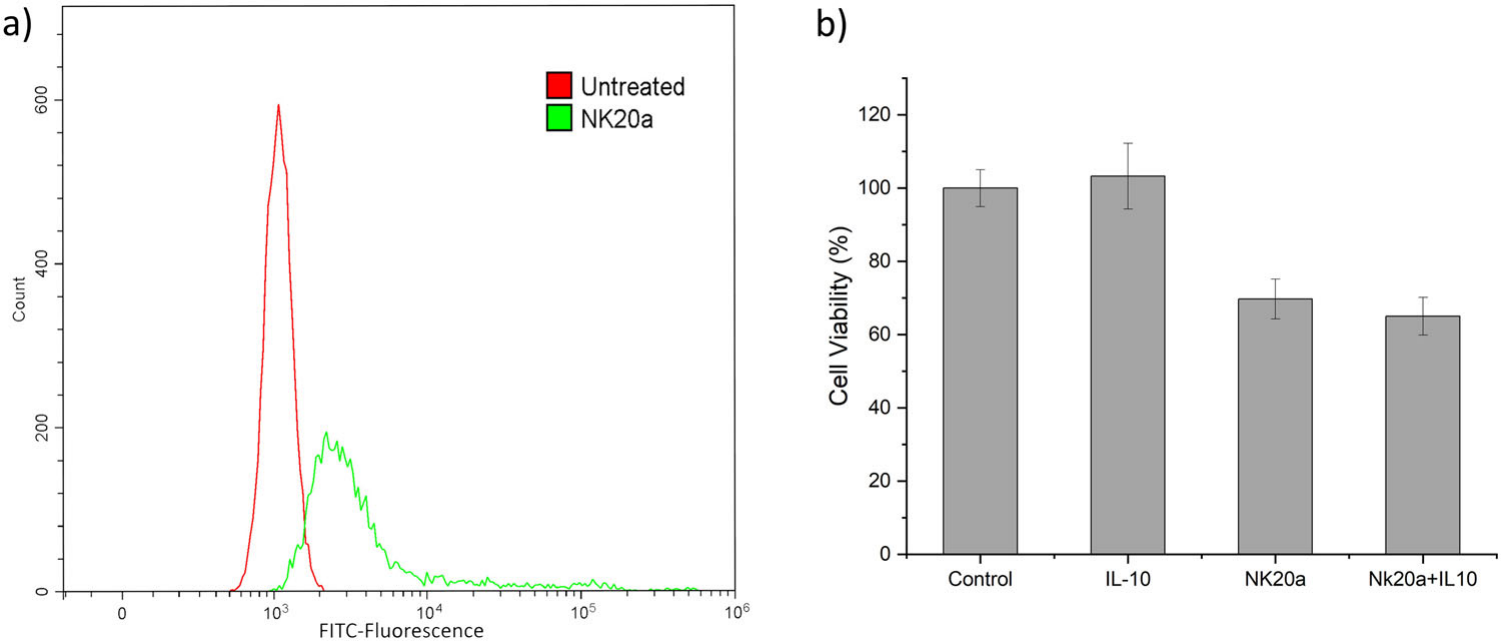
In vitro binding assay and cytotoxicity study. **a** B-lymphoma cells were treated with NK20a, and the fluorescence of the treated cells was analyzed through flow cytometry to determine the binding ability of NK20a to the cell surface. The fluorescence distribution of FITC was categorized as treated with NK20a (green) and untreated (red). **b** Bar chart showing the cell viability of the B-lymphoma cell line treated with 10 ng/mL IL-10, 50 μg/mL NK20a, and both, respectively. Error bars represent the standard deviation of experiments done in triplicate.

Studies have suggested that IL-10 is overexpressed in several cancer cells, which can change the tumor microenvironment to enable tumor cells to easily escape from the immune system^11,32^. To assess the binding effect of NK20a on the cancer cell surface, BJAB cells were treated with NK20a labeled with the fluorescent dye FITC. The fluorescence of the treated cells was detected through flow cytometry. The results revealed that the fluorescence of the cells treated with NK20a was elevated compared with untreated cells (Fig. 4a), indicating that the fluorescent NK20a peptide was able to bind to the surface of BJAB cells. We also investigated the effects of NK20a on BJAB cell proliferation and found that the proliferation of BJAB cells was inhibited when the cells were exposed to 50 μg/mL NK20a. An approximately 30% reduction in cell proliferation was measured in the peptide-treated group compared with the untreated control group (Fig. 4b). Similar results were observed in the NK20a+IL-10 group, indicating that when the cells were treated with NK20a, the addition of IL-10 cytokine did not reverse the effects of NK20a. In this experiment, IL-10 cytokine incubation alone did not significantly promote the proliferation of lymphoma B cells, as the B cells themselves could produce endogenous IL-10. We suspected that the cells were already in their optimized growth conditions. The additional IL-10 could not significantly promote the proliferation of the cells. However, as IL-10 can only function through binding to the cell surface receptors for triggering signal transductions in the cells^33^, blocking the receptors should affect cell growth or survival, even with the presence of endogenous IL-10. As could be seen in this experiment, the peptide treatment, which aimed to block the IL-10 receptor on the cell surface, was able to significantly reduce the viability of lymphoma B cells. In our BLI kinetic assays and flow cytometric analysis, it has been confirmed that peptide NK20a can bind to the recombinant receptor human IL-10Ra protein and can bind onto the BJAB cell surface. In this experiment, when the cells were treated with NK20a, the reduction of cell viability could not be rescued by adding extra IL-10, further implying that IL-10 receptors on the cell surface were blocked by NK20a.

### NK20a can inhibit the anti-inflammatory effects of IL-10 on LPS-stimulated macrophage cells

**Fig. 5:**
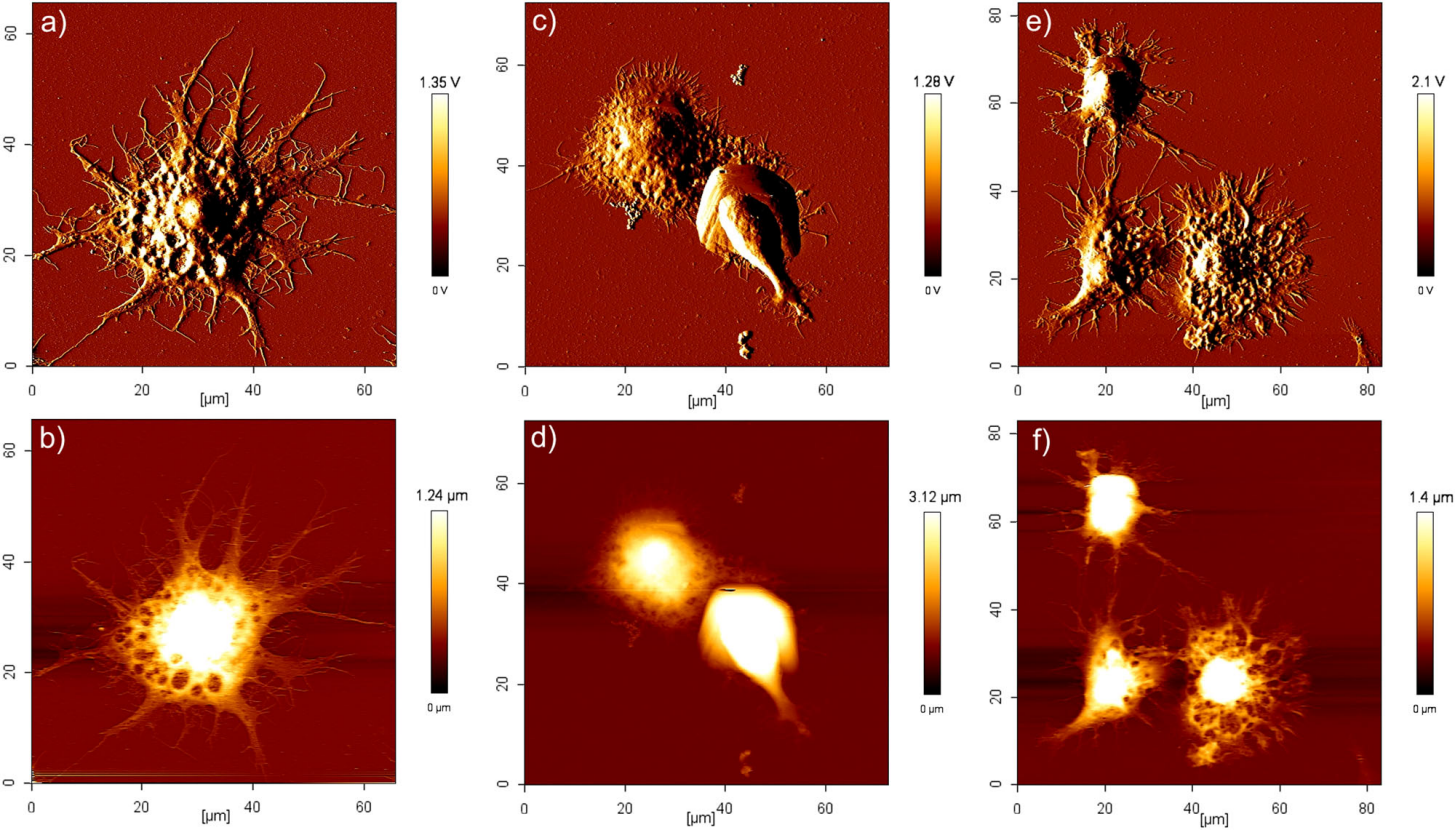
Morphological changes of RAW 264.7 macrophages observed through AFM showing inhibition of immunosuppression and maintenance of inflammatory response. **a**, **b** Morphology of a RAW 264.7 cell showing inflammatory state after treatment with 10 ng/mL LPS. As shown in the images, numerous pseudopodia displayed unbridled spreading from the cell body. **c**, **d** Morphology of a RAW 264.7 cell cotreated with LPS (10 ng/mL) and IL-10 (10 ng/mL). As shown in the images, IL-10 inhibited the inflammation of LPS-stimulated cells, and the cells became round and plump in shape. **e**, **f** RAW 264.7 cells were pretreated with LPS (10 ng/mL) and NK20a (50 μg/mL) for 30 min, followed by exposure to IL-10 (10 ng/mL). As shown in AFM images, NK20a indeed blocked the IL-10 receptor and thus inhibited the immunosuppression caused by IL-10 and maintained cell inflammation.

IL-10 has been shown to suppress immune response, and this immunosuppression helps tumor cells to evade host immune clearance, thereby promoting proliferation and metastasis of cancer cells^10,34^. Macrophages play a key role in tumor cell clearance. IL-10 has been suggested to inhibit macrophage functions^5^. In this study, we examined the ability of NK20a to inhibit the anti-inflammatory effects of IL-10 on macrophages. The RAW 264.7 macrophage cell line was used in the evaluation, and the activation of macrophage cells was visualized through AFM. We treated the cells with the bacterial endotoxin LPS (10 ng/mL) to stimulate the activation of macrophage cells. As evident in Fig. 5a, b, upon activation by LPS, the macrophage cells appeared to be extended and have an increased number of pseudopodia. When the cells were cotreated with LPS and IL-10 (10 ng/mL), the activation of macrophages was suppressed (Fig. 5c, d). To explore the ability of NK20a to inhibit the anti-inflammatory effects induced by IL-10, we pretreated the cells with NK20a (50 μg/mL) and LPS (10 ng/mL) for 30 min and then treated them with IL-10 (10 ng/mL). After NK20a pretreatment, IL-10 was unable to suppress the activation of the RAW 264.7 macrophage cells (Fig. 5e, f). Thus, NK20a can bind to the IL-10 receptor and abolish the effects of IL-10 on macrophage cells.

### Characterization of NK20a-conjugated gold nanoparticles

**Fig. 6:**
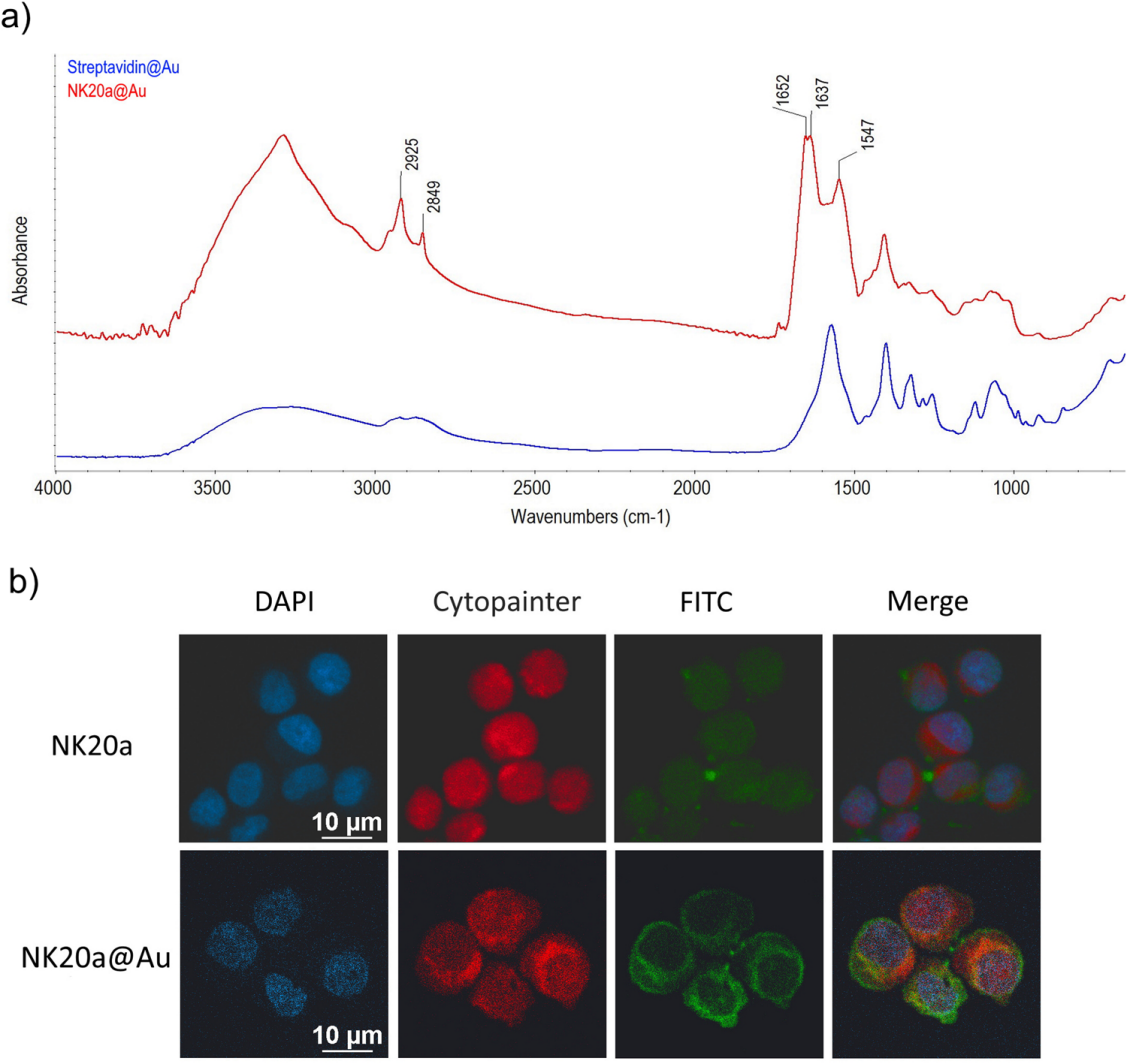
FTIR spectrum of NK20a@Au nanoparticles and validation of gold particles as drug delivery materials through in vitro confocal microscopy. **a** Representative absorption FTIR spectra of streptavidin@Au binding to biotinylated NK20a (red line) compared with the spectra of streptavidin@Au surface (blue line). Typical vibration of biotin-block streptavidin was observed at peaks located at 1637 and 1547 cm^−1^. The peak at 1652 cm^−1^ represents the NK20a amide I band. The peaks at 2849 and 2925 cm^−1^ are related to the stretching vibration of C–H and N–H bonds, respectively. **b** B-lymphoma cells were incubated with NK20a@Au and NK20a at room temperature for 20 min. Images were taken after the cells were fixed in glutaraldehyde for 20 min and then washed with 1× PBS. The cell nuclei were counterstained with DAPI staining (blue). The cell membrane was stained with Cytopainter (red). NK20a was labeled with FITC (green). Merged images represent the nuclei, cell membrane, and NK20a locations. NK20a single staining and merged images show that the peptide could bind to the IL-10 receptor on the plasma membrane as indicated by Cytopainter colocalization with FITC fluorescence. NK20a@Au fluorescence was distributed in the cytoplasm but not in the nuclei and cell membrane; FITC fluorescence was not observed in the nucleus area. These results indicate that NK20a@Au successfully penetrated the membrane into the cytoplasm through cell uptake. Scale bar: 10 µm.

Conjugating peptides to nanoparticles is a favorable strategy for peptide delivery and increasing peptide in vivo stability^35^. Gold nanoparticles are a popular choice for conjugating antibodies, targeting molecules for cancer, and therapeutic molecules because of their bioavailability, chemical inertness, and minimal toxicity^36,37^. In our study, gold nanoparticles were used as carriers for our functionalized peptide NK20a. We examined the pharmacological activity of NK20a conjugated onto gold nanoparticles (NK20a@Au). FTIR spectroscopy was used to confirm the conjugation of the peptide onto the gold nanoparticles. Figure 6a presents the FTIR spectra of streptavidin-conjugated functionalized gold nanoparticles (streptavidin@Au) and NK20a@Au. The peaks at 1637 and 1547 cm^−1^ in the NK20a@Au spectrum correspond to the streptavidin–biotin complex^38^, and the peak at 1652 cm^−1^ represents the amide I band of peptide bonds, which is considered characteristic of the backbone of NK20a. The peaks at 2849 and 2925 cm^−1^ were related to C–H and N–H stretching vibrations, respectively. The peptide characteristic peaks observed in the NK20a@Au spectrum indicated that NK20a was indeed conjugated onto gold nanoparticles.

### Cellular distributions of NK20a-conjugated gold nanoparticles

The cellular distribution of NK20a@Au in the treated cells was examined through confocal fluorescence microscopy. In this study, DAPI with blue fluorescence was used as the cell nucleus dye, Cytopainter with red fluorescence was used as the cell membrane probe, and NK20a peptide was labeled with FITC. The confocal microscope images of B-lymphoma cells under different treating conditions are presented in Fig. 6b. The images show that the distribution of NK20a was similar to that of biological membranes, indicating that NK20a interacted with and bound to the cell membrane. However, in the group treated with NK20a@Au, green fluorescence, representing the locations of the peptide, was distributed in the whole cell except the nucleus. These results imply that NK20a@Au was able to enter the cells and was distributed in the cytosol; we propose that NK20a can interact with IL-10Ra on the cell surface, and the interaction can deliver gold nanoparticles to the cells, allowing the cell uptake of the nanoparticles.

### Therapeutic potential of NK20a-conjugated gold nanoparticles coloaded with the chemotherapeutic drug 5-FU

We functionalized the gold nanoparticles with Poly A15, a polynucleotide with 15 adenines. Poly A15 was used to conjugate 5-FU to enable loading of the gold nanoparticles with 5-FU. As demonstrated previously, conjugation with NK20a could facilitate cell entry of the gold nanoparticles. If NK20a@Au could also be loaded with 5-FU, NK20a@Au can be used as a material for delivering the cytotoxic therapeutic drug to cancer cells. In this study, we co-conjugated NK20a and Poly A15 onto gold nanoparticles with streptavidin–biotin interactions. According to DLS measurements (Fig. S4A, S4B), the average particle size of streptavidin@Au in Milli Q water was 91.5 nm, and the polydispersity index was 0.247. After the nanoparticles were functionalized with NK20a and Poly A15 (NK20a/Poly A 15@Au), the average particle size was 542 nm, and the polydispersity index was 0.463 (in PBS). The average zeta potential for streptavidin@Au in Milli Q water was −41.98 ± 1.65 mV, and that for NK20a/Poly A15@Au in PBS was −37.67 ± 1.33 mV (Fig. S4C). The streptavidin gold nanoparticles were initially negatively charged; upon surface functionalization with the peptide, the measured particle size increased, and the polydispersity index slightly changed because the positive charges contributed by NK20a neutralized the charges of the nanoparticle surface, reducing the repulsion between NK20a@Au nanoparticles, and increasing the possibility of aggregation. These effects were also observed in TEM images (Fig. S5A), in which the nanoparticles of NK20a@Au tended to aggregate more (red arrows in Fig. S5A), as compared to NK20a/Poly A15@Au (Fig. S5B). Interestingly, in the presence of the Poly A sequence, the negatively charged polynucleotides could contribute more negative charges to the particles, resulting in an increase in the negative zeta potential and repulsion between particles. The conjugation of Poly A was able to provide greater stability of dispersed gold nanoparticles (Fig. S5B). Although the particle size increased after functionalization, the zeta potential did not significantly change, and NK20a/Poly A15@Au still had good solubility and stability.

**Fig. 7:**
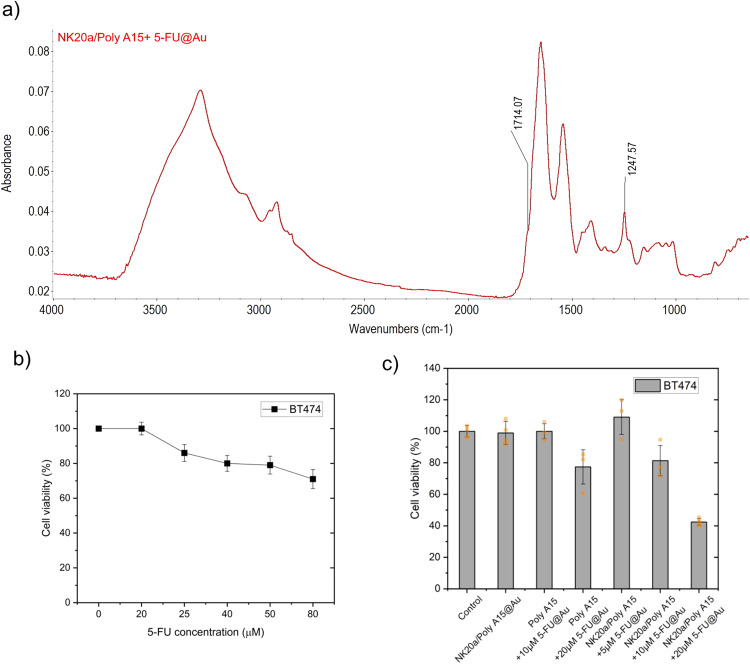
FTIR spectrum of synthesized NK20a/Poly A15@Au + 5-FU nanoparticles and therapeutic effect of 5-FU and gold nanoparticles on breast tumor cells. **a** The loading of 5-FU on NK20a/Poly A15@Au nanoparticles was confirmed. The peak at 1247 cm^−1^ on the FTIR spectrum corresponded to 5-FU C–F stretching vibrations, and the peak at 1714 cm^−1^ corresponded to 5-FU with base adenine pairing. **b** Cell viability was determined using the CCK-8 assay after exposure to increasing concentrations of 5-FU for 72 h. **c** Cells incubated with NK20a/Poly A15@Au; Poly A15@Au containing 5-FU with equivalent concentrations of 10 and 20 μM; and NK20a/Poly A15@Au containing 5-FU with equivalent concentrations of 5, 10 and 20 μM. The dots represent individual data points, illustrating data distribution. Error bars indicate the standard deviation from experiments conducted in triplicate.

After NK20a/Poly A15@Au nanoparticles were fabricated, 5-FU was loaded onto them through nucleotide base pairing. In the FTIR spectrum of the 5-FU-loaded NK20a/Poly A15@Au nanoparticles (Fig. 7a), the peak at 1247 cm^−1^ belonged to the carbon-fluorine stretching band of 5-FU, and the peak at 1714 cm^−1^ corresponded to the 5-FU with base adenine pairing. These peaks in the FTIR spectrum indicated that 5-FU was indeed loaded onto NK20a/Poly A15@Au nanoparticles. In this experiment, the concentrations of gold nanoparticles and ligands were calculated based on the Beer–Lambert law, and the results are presented in Table 3. In this study, we used the gold nanoparticle concentration of 3.4 × 10^−9^ M in each test.

**Table 3:** Extinction coefficients (ε) of gold nanoparticles with functionalized ligands and molar gold NP concentration (in mol/L).

Name	Concentration (mol/L)	Core size (nm)	ε (M^−1^ cm^−1^)
Au	3.4 × 10^-9^	40	8 × 10^9,a^
Name	Mw (Da)	λ(nm)	ε **(M**^**−1**^ **cm**^**−1**^**)**
Biotin-Poly A15	5032	260	205,200^b^
Biotin-NK20a	2728	205	87,930^c^
5-FU	130	266	6730^d^

^a^Extinction coefficient obtained from the literature^54^.

^b^The extinction coefficient is calculated as follows: ε260 = ((Sum of ε260 for all bases) + (ε260 for all modifications)) × 0.9, to adjust for hyperchromicity. It can be calculated online at https://www.biosearchtech.com/oligospec-calculator-6628.

^c^Extinction coefficients at 205 and 260 nm can be calculated based on the sequence^55^.

^d^Molar extinction coefficient of 5-FU in aqueous solution at 266 nm was obtained from the literature^56^.

### Therapeutic potential of 5-FU-loaded NK20a/Poly A15 double-conjugated gold nanoparticles on breast cancer cells

5-FU is a cytotoxic chemotherapeutic drug used in the treatment of a variety of cancers, including breast cancer^39^. However, its clinical applications are limited by several drawbacks, such as its nonspecific toxicity, poor membrane permeability, and short half-life in plasma^40^. The gold nanoparticles fabricated in this study may be used as a delivery material to deliver 5-FU into cancer cells. Here, we loaded 5-FU onto NK20a/Poly A15@Au and Poly A15@Au nanoparticles. In the concentration of gold particles with an optical density (OD) of 1.0 at 530 nm, the maximum 5-FU loading capacity for NK20a/Poly A15@Au and Poly A15@Au was approximately 20 μM and 30 μM, respectively (according to OD values at 266 nm for 5-FU^41^, Table S1). The BT-474 breast cancer cells were treated with free form 5-FU, NK20a/Poly A15@Au, 5-FU- loaded Poly A15@Au or 5-FU-loaded NK20a/Poly A15@Au for 72 h, and the therapeutic effects in these treatment groups were measured using the CCK-8 assay. The results are shown in Fig. 7b, c; According to Fig. 7b, 5-FU alone did not exhibit a significant therapeutic effect against the breast cancer cells. Even when the 5-FU concentration was increased to 80 μM, the cancer cells still maintained a survival rate of more than 70%. In the cases of 5-FU-loaded nanoparticle treatments, it was found that the treatment of Poly A15@Au loaded with 10 μM 5-FU did not significantly affect cell survival. However, when the loaded 5-FU concentration was increased to 20 μM, the treatment resulted in a 22% reduction in cell survival of breast cancer cells (Fig. 7c). On the other hand, the viability of the cancer cells treated with 5-FU-loaded NK20a/Poly A15@Au nanoparticles at an equivalent 5-FU concentration of 20 μM was significantly reduced to a level lower than 45%, and the reduction was in a dose-dependent manner (Fig. 7c). Thus, the therapeutic effect of 5-FU was greatly enhanced by the conjugation of the drug with NK20a@Au nanoparticles.

## Discussion

In this study, we refined the original in silico–designed peptide NM25^21^ derived from the cytokine IL-10 to increase its binding affinity to the receptor subunit IL-10Ra. Two peptides, NK20 and NK20a, were designed through amino acid truncation and substitutions based on the bioinformatic analysis of the binding interface of the IL-10/IL-10Ra complex structure. According to the binding free energy calculations, of the three peptides (NM25, NK20, and NK20a), NK20a had the highest binding affinity to IL-10Ra. This in silico calculation was validated experimentally by using the BLI assay, which also showed that NK20a had the highest affinity to IL-10Ra protein. The improvement in the binding of the peptide to IL-10Ra from NM25 to NK20 (five amino acids truncated at the C-terminal of NM25) was significant. The association constant increased substantially from 8.32 × 10^2^ to 1.02 × 10^4^. To reduce the number of negative charges and increase the association with IL-10Ra, we further replaced two aspartic acid (D) residues with asparagine (N) to yield NK20a. After the amino acid replacements, the dissociation constant decreased from 0.2185 for NK20 to 0.09 for NK20a, indicating stronger binding between the peptide and the receptor. Our results clearly demonstrated that NK20a had the highest binding affinity to IL-10Ra, and our refinement was successful. The MM/PBSA binding free energy calculations for these three designed peptides revealed that compared with the original peptide NM25, the peptide NK20a with two amino acid mutations had lower polar solvation energy and higher electrostatic energy, which led to higher binding affinity to IL-10Ra (Fig. 2).

As mentioned, IL-10 might play different roles in various cancers. IL-10 affects various types of cancer cells differently^3^. For example, B-lymphoma cells depend on the IL10-STAT3 signaling pathway for survival and proliferation. The effect of IL-10 on cell survival promotes IL-6 expression and synthesis, which promotes cell proliferation through the upregulation of B-cell lymphoma-2 (Bcl-2) expression and a shift of the proliferation/apoptosis balance toward neoplastic cell proliferation^10,42^. Blockade of the IL-10 receptor results in cancer cell death and can possibly be a stand-alone therapeutic treatment for cancers^43–45^. In our study, we designed an NK20a peptide with excellent specific affinity to IL-10Ra, the ligand binding subunit of the IL-10 receptor. Treatment with this peptide blocked the IL-10 receptor and reduced the number of viable B-lymphoma cells (Fig. 4b). This result is consistent with the belief that blockade of the IL-10 receptor inhibits cell proliferation and survival.

Macrophages are elements of a host’s immune system that defend the host from the invasion of pathogens. They also play a major role in abnormal or cancer cell clearance. IL-10 is overexpressed in patients with a variety of cancers^1,12–14^, and elevated IL-10 expression is correlated with worse outcomes in cancer patients^46^. IL-10 has been indicated to suppress macrophage functions, leading to cancer cells’ evasion of host clearance^5^. Our designed peptide was tested for the ability to inhibit this effect of IL-10 on macrophages and maintain the activated status of the stimulated macrophages. Our designed peptide might also be used as a synergetic agent to assist the immune system in clearing cancer cells in patients with cancer.

5-FU is one of the most commonly used chemotherapeutic drugs to treat breast cancer. It works by inhibiting the normal synthesis of thymidine by incorporating 5-FU metabolites into DNA and RNA, eventually leading to the arrest of synthetic pathways and subsequent apoptosis of treated cells^47,48^. 5-FU is typically administered via intravenous injection. Infusions of 5-FU can be rapidly absorbed through the circulatory system, and prolonged administration of 5-FU is required to maintain a high level of 5-FU in the body, thereby enhancing clinical efficacy. However, its nonspecific toxicity, poor membrane permeability, and a very short half-life of only 8 to 20 min in plasma may lead to adverse side effects and low therapeutic efficiency^49^, which limit the therapeutic applications of 5-FU^50^. In the present study, we designed and fabricated NK20a/Poly A15@Au nanoparticles for possible anticancer drug delivery. We applied the specific binding property of NK20a to the IL-10 receptor to target the 5-FU-loaded nanoparticles to the cancer cell surface and distribute the 5-FU into the cells through endocytosis of the nanoparticles. This strategy enhanced the therapeutic effect of 5-FU on breast tumor cells (Fig. 7). Moreover, the proliferation of the breast cancer cell line BT-474 was not affected by direct treatment with NK20a, indicating that IL-10 might not directly be involved in the proliferation and survival pathways of breast cancer cells. The NK20a/Poly A15@Au nanoparticles exerted their effect by synergistically releasing chemotherapy drugs to inhibit cell growth. Regarding drug-release efficiency, our results were similar to those reported in previous studies that reported 5-FU release rates from nanoparticles of 28%–38% at pH 7.4 and 37 °C within 24 h^51,52^. In addition, an acidic extracellular pH is a vital feature of tumor tissue associated with tumor metastasis^53^. The low-pH environment also provides a condition for efficient 5-FU release^52^.

## Conclusions

Based on in silico molecular simulations and theoretical calculations, NK20a, a peptide derived from IL-10 with improved affinity toward the ligand binding subunit of the IL-10 receptor IL-10Ra, was designed. The binding ability of the in silico–designed peptide to the receptor was validated experimentally through a BLI assay. The peptide exhibited the ability to bind to the IL-10 receptor–expressing cells and inhibited the growth of B-lymphoma cells. This peptide may also potentially prevent functional suppression of activated macrophages by IL-10 and could maintain the activated status of the macrophages. NK20a can also be used to functionalize gold nanoparticles for cell targeting and anticancer drug delivery. The conjugation of the peptide with gold nanoparticles can facilitate the cellular targeting of nanoparticles through endocytosis. We also loaded the anticancer drug 5-FU to the peptide-conjugated gold nanoparticles and found that the loading on the nanoparticles enhanced the efficacy of 5-FU in the treatment of BT-474 breast cancer. With modifications, NK20a-conjugated gold nanoparticles have the potential for use in the delivery of other anticancer therapeutics. This study demonstrated that peptides can be used not only as direct or indirect treatments for cancer but also as navigators to guide drug carriers to target cells.

### Supplementary information

Supplementary Information

Reporting Summary

## Data Availability

The data that support the findings of this study are available from the corresponding author upon reasonable request. The initial structure files and MD trajectories used in the study are deposited in the persistent repository, figshare (10.6084/m9.figshare.24635289.v1).
