# Supplementary material for: A peptide derived from interleukin-10 exhibits potential anticancer activity and can facilitate cell targeting of gold nanoparticles loaded with anticancer therapeutics
Source: Commun Chem. 2023 Dec 15;6:278. doi: 10.1038/s42004-023-01079-x (PMC10724200; doi:10.1038/s42004-023-01079-x)
Supplement: Supplementary file 1 — Supplementary Information [file 42004_2023_1079_MOESM1_ESM.pdf]

Supporting Information

A peptide derived from interleukin-10 exhibits potential anticancer activity and can facilitate cell targeting of gold nanoparticles loaded with anticancer therapeutics

Chun-Chun Chang[1,2,$], Chin-Hao Yang[3$], Chin-Hsien Chuang[4], Shinn-Jong Jiang[3], Yin-Min Hwang[2], Je-Wen Liou[2,3*] and Hao-Jen Hsu[3,4*]

[1]*Department of Laboratory Medicine, Hualien Tzu Chi Hospital, Buddhist Tzu Chi Medical Foundation, Hualien 97004, Taiwan*
[2]*Department of Laboratory Medicine and Biotechnology, College of Medicine, Tzu Chi University, Hualien 97004, Taiwan*
[3]*Department of Biochemistry, School of Medicine, Tzu Chi University, Hualien 97004, Taiwan*
[4]*Department of Biomedical Sciences and Engineering, College of Medicine, Tzu Chi University, Hualien 97004, Taiwan*
[$]*These authors contributed equally to this work*

Correspondence:

*Hao-Jen Hsu: e-mail address: hjhsu32@mail.tcu.edu.tw

*Je-Wen Liou: e-mail address: jwliou@mail.tcu.edu.tw

Tel: +886-3-8565301 ext. 2643. Fax: +886-3-8572526

Keywords: Computational design; Functional peptide; IL-10; Anticancer; Nanoparticles

(A)

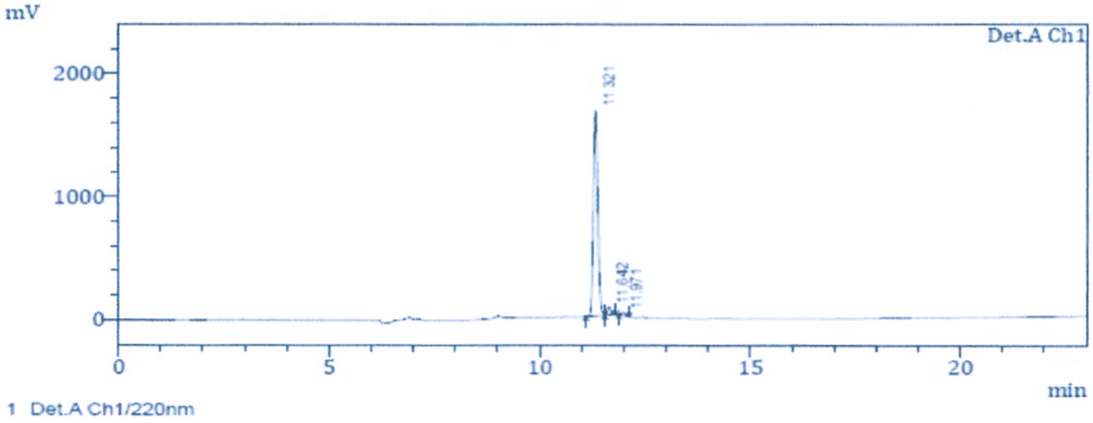

PeakTable

Detector A Ch1 220nm

Peak#	Ret. Time	Area	Height	Area %	Height %
1	11.321	12310850	1673433	95.060	94.752
2	11.642	456879	68221	3.528	3.863
3	11.971	182923	24459	1.412	1.385
Total		12950652	1766113	100.000	100.000

(B)

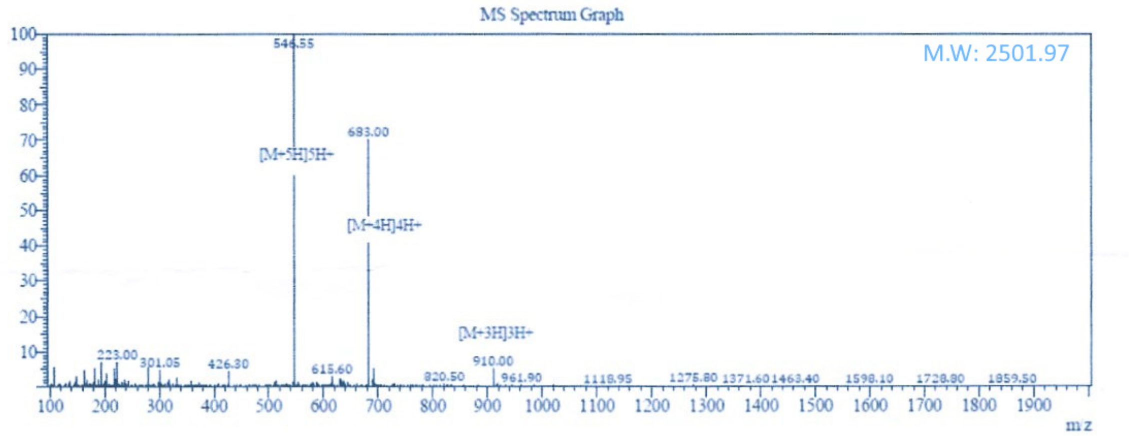

Fig. S1: Representative results of peptide purity evaluation. The purity of peptides was confirmed through HPLC and ESI mass spectrometry. (A) HPLC analysis of purified NK20a shows that there was only one major peak at retention time of 11.321 min in the chromatogram, and the area analysis of the peaks indicates the purity of peptide was over of 95%. (B) Confirmation of the peptide identity in the peak with a retention time of 11.321 min in (A) by using mass spectrometry.

(A) **(B)**

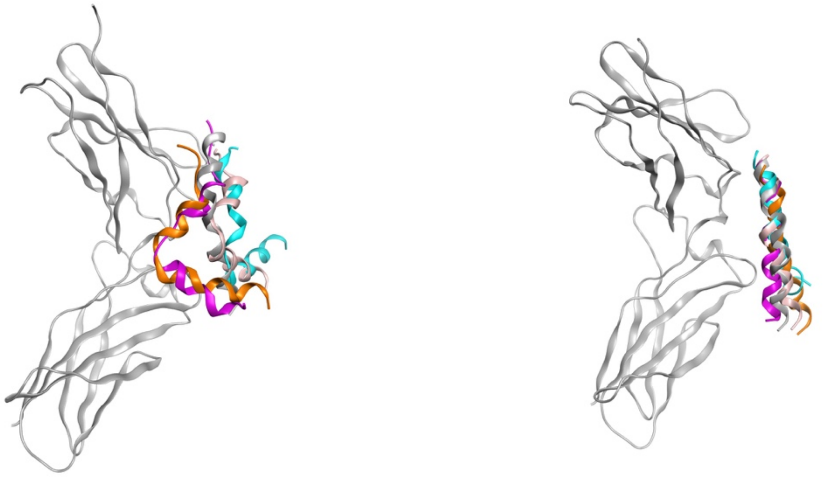

Fig. S2: Snapshots of peptides bound to receptor IL-10Ra at various simulation times. (A) NM25 binding to IL-10Ra (B) NK20a binding to IL-10Ra. The subunit receptor IL-10Ra is shown in gray color; peptides at various simulation times: gray color (0 ns); skin color (5 ns); cyan color (10 ns); orange color (25 ns); purple color (50 ns).

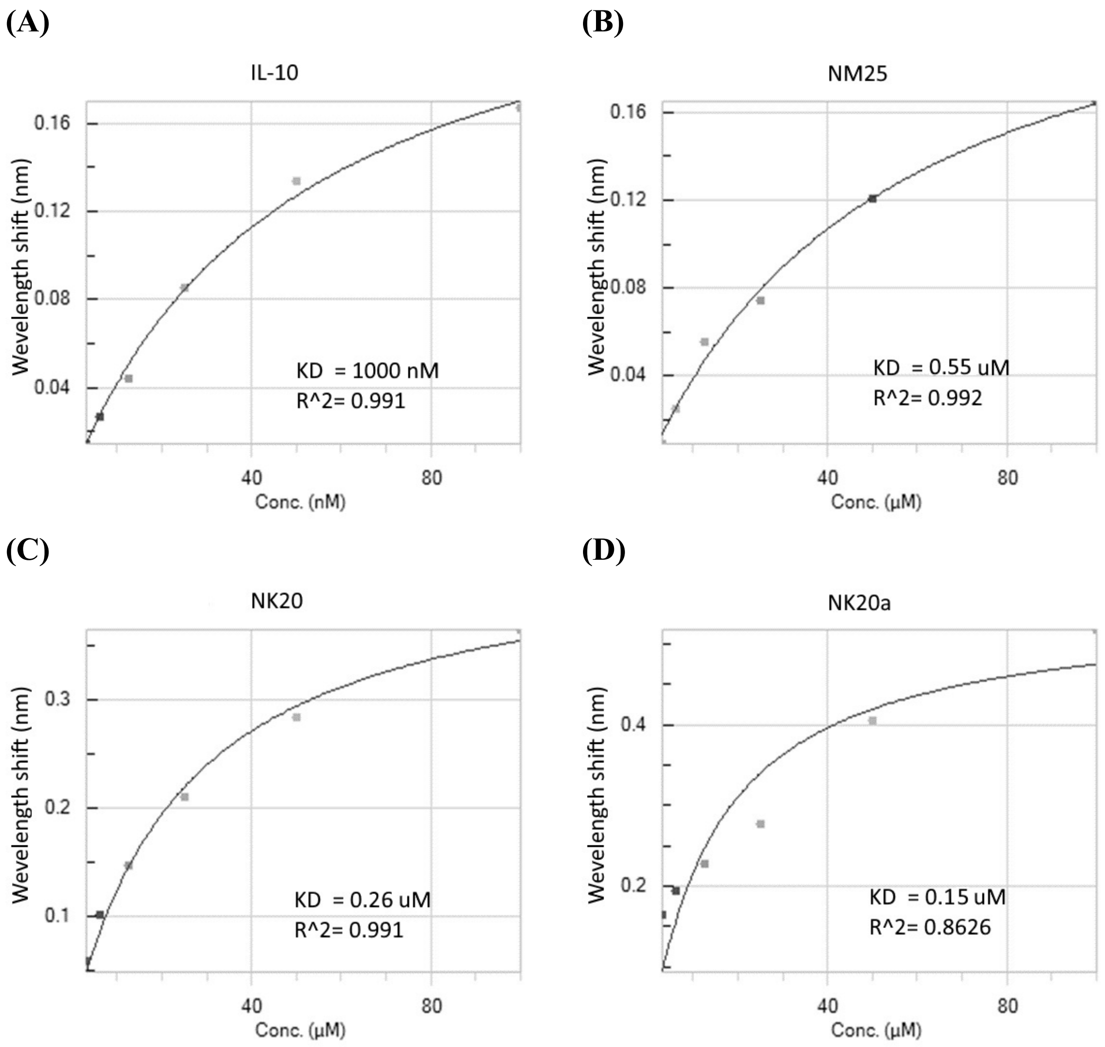

Fig. S3: Binding affinities of peptides/protein to IL-10Ra determined using steady-state analysis. K_D values for (A) IL-10, (B) NK25, (C) NK20, and (D) NK20a were measured to be 1.00, 0.55, 0.26 and 0.15 μM, respectively.

(A)

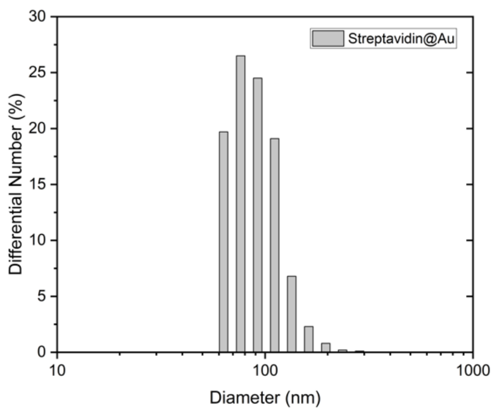

(B)

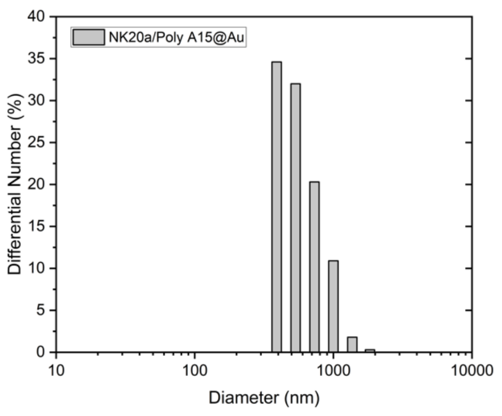

(C)

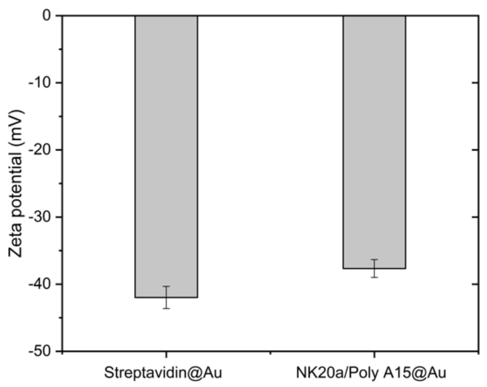

Fig. S4: DLS and zeta potential analysis results for the gold nanoparticles. (A) DLS hydrodynamic diameter measurement of streptavidin@Au resolubilized in distilled water. (B) DLS hydrodynamic diameter measurement of NK20a/Poly A15@Au resolubilized in PBS buffer. (C) Surface zeta potential of the gold nanoparticles. Error bars represent standard deviation of the measurements of each system.

(A)

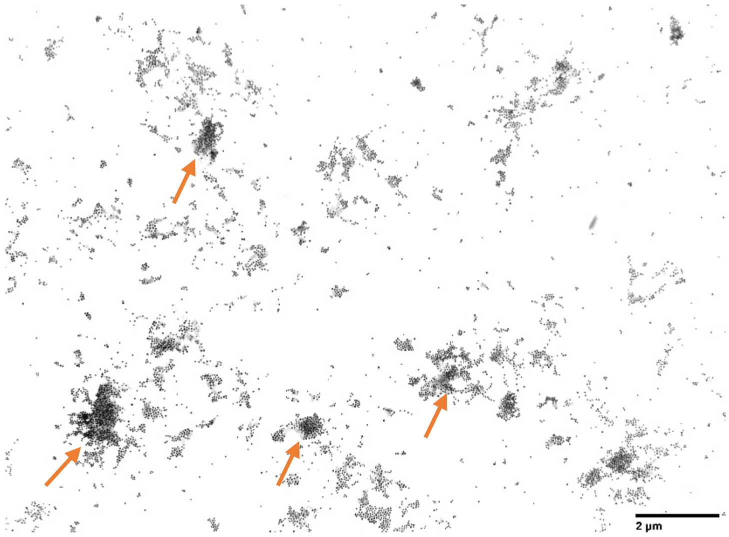

(B)

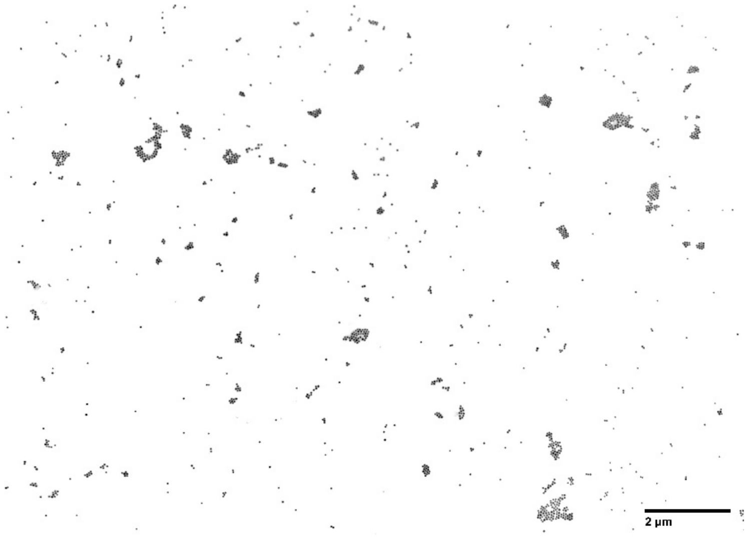

Fig. S5: Transmission electron microscopy (TEM) images of NK20a-conjugated gold nanoparticles and NK20a/Poly A15 double-conjugated gold nanoparticles. (A) NK20a-conjugated gold nanoparticles. (B) NK20a/Poly A15 double-conjugated gold nanoparticles. The red arrows indicate large particle aggregations. The scale bar in each image represents 2 μm.

**Table S1. 5-FU loading capacity for NK20a/Poly A15@Au and Poly A15@Au**		
Name	5-FU optical density	5-FU Concentration (mol/L)
Poly A15@Au	0.1912 ± 0.0143	30.21 ±2.27
NK20a/Poly A15@Au	0.1320 ± 0.0101	20.83 ±1.54

The measurements were conducted with the same gold concentration of an optical density of 1.0 at 530 nm. 5-FU loading capacity was calculated based on the optical absorbance at 266 nm, and the Beer–Lambert law with the extinction coefficient of 6730 M^{-1} cm^{-1} for 5-FU with a 10 mm path length.
